# Anti-amyloid therapies: are they effective and safe?

**DOI:** 10.1192/j.eurpsy.2024.72

**Published:** 2024-08-27

**Authors:** R. Perneczky

**Affiliations:** Department of Psychiatry and Psychotherapy, LMU Hospital, LMU Munich, Munich, Germany

## Abstract

**Abstract:**

After numerous unsuccessful attempts to create a therapy that could alter the course of Alzheimer’s disease, first monoclonal antibodies targeting amyloid-β in the brain have finally shown consistent evidence of clinical effectiveness. These therapies not only slow the progression of the disease, but also show positive results in secondary clinical outcomes and reduced amyloid-β levels on PET scans. This presentation will examine the main features of the previous failed trials and explore possible reasons for their lack of success in developing a treatment for early-stage Alzheimer’s disease. It will also compare the safety profiles of various antibodies and point out precautions that should be taken when using them in regular clinical practice. Furthermore, it will be discussed how blood-based biomarkers can revolutionize the clinical care pathway, making it easier to adopt antibody treatments. A comprehensive model that integrates case-finding and treatment across various healthcare sectors will be proposed. In conclusion, we may have made a significant breakthrough by demonstrating that reducing amyloid-β levels leads to clinical benefits, not just changes in biomarkers. As the new generation of drugs becomes more commonly used, we will see whether their statistical effectiveness translates into meaningful clinical changes. This could mark the start of a new phase in the development of drugs for Alzheimer’s disease.

**Disclosure of Interest:**

None Declared

